# The Book World of Medicine and Science

**Published:** 1896-09-05

**Authors:** 


### The Book World of Medicine and Science.

Infantile Mortality during Childbirth, and its Prevention. By A. Brothers, B.S., M.D. The Furness Jenks Prize Essay of the College of Physicians of Philadelphia, (Philadelphia: P. Blakiston, Son, and Co. London: Kegan Paul, Trench, Trübner, and Co. 1896. Price 7s. 6d. nett.)

The importance of the subject dealt with in this volume is best shown by quoting a few figures. The total number of births which took place in New York City in 1892 amounted to 49,447. In the same year there were 3,573 still births, of which 1,539 were still births at full term; and, of children born alive, 2,814 died within four weeks of their birth, many, no doubt, from conditions arising in connection with their entry into the world. If, then, one adds together the still births and the cases in which the infants, although born, failed to live a month, one easily sees what a mass of procreative effort is wasted, and what an amount of pain and suffering is borne for naught. The author deals first with the maternal causes (*a*) preceding and (*b*) during labour, and then in the same way deals with fœtal causes (*a*) preceding, (*b*) during, and (*c*) following labour. Needless to say, the field is a large one, ranging over such subjects as tuberculosis, cholera, lead poisoning, placenta previa, the whole range of deformities, and of the causes of difficult and obstructed labour, with the various operations for their relief, and over almost every malady and accident that can happen to the fœtus and the child. Naturally, then, the book is a discursive one, and here and there runs rather into catalogue form; but it is a good presentment of a very large question, and shows well how wide should be the knowledge of those who superintend the complex processes involved in childbirth.

Sterility. By Robert Bell, M.D. (London: J. and A. Churchill. 1896. Price 5s.)

This book is sufficiently dogmatic to render it at least easy reading; at the same time it is sufficiently positive in its hopefulness, and in its assertions that a sterile woman is a diseased one to make it rather dangerous reading if left about, for women are easily led astray by foolish fancies. The gist and scope of Dr. Bell's treatise may be gathered from a few quotations. "It, however, being my object to confine my attention specially to the subject of sterility in the female, with whom, in the large majority of cases, the fault usually lies, I may proceed to observe that I consider the one great factor in its production to be a diseased condition of the endometrium." "If pregnancy does not follow marriage within a reasonable time, there must of necessity, in the majority of cases, be present some unhealthy condition of the generative organs of the female." "The future of the patient, however, is not desperate if the endometrium, by judicious treatment, is restored to its normal condition, and I have no hesitation in affirming that when this is accomplished—no matter how long after marriage—the most powerful, as well as the most frequent, barrier to conception is removed." The other side of the question, however, is thus presented: "The more extensive my experience the firmer becomes my conviction that endometritis is the one potent cause of sterility, and not only of sterility, but of various other affections of the uterus, amongst which the most important are flexions, and . . . in the large majority of instances disease both of the tubes and ovaries is secondary to this also." And what is the universal remedy for all these ills, the etiology of which appears so simple to Dr. Bell? The treatment he "invariably" commences by curetting, after which he applies iodised phenol once and a tampon twice a week, and in two to four months recovery is complete! We do not like the book; we doubt such simple pathology; we do not feel drawn to doctors whose treatment is so invariable; and we have but small faith in the curette, which he depicts as a straight instrument, which he appears to twirl round within the uterus. Fortunately there are still many women in the world who are both healthy and happy though childless.

